# Myopia and Nutrient Associations with Age-Related Eye Diseases in Korean Adults: A Cross-Sectional KNHANES Study

**DOI:** 10.3390/nu16091276

**Published:** 2024-04-25

**Authors:** Jeong-Mee Kim, Yean-Jung Choi

**Affiliations:** 1 Department of Visual Optics, Far East University, Eumseong 27601, Republic of Korea; jmkim@kdu.ac.kr; 2 Department of Food and Nutrition, Sahmyook University, Seoul 01795, Republic of Korea

**Keywords:** myopia prevalence, age-related eye diseases, Korea national health and nutrition examination survey (KNHANES), cataracts, glaucoma, macular degeneration

## Abstract

This study assessed the prevalence of myopia, cataracts, glaucoma, and macular degeneration among Koreans over 40, utilizing data from the 7th Korea National Health and Nutrition Examination Survey (KNHANES VII, 2018). We analyzed 204,973 adults (44% men, 56% women; mean age 58.70 ± 10.75 years), exploring the association between myopia and these eye diseases through multivariate logistic regression, adjusting for confounders and calculating adjusted odds ratios (ORs) with 95% confidence intervals (CIs). Results showed a myopia prevalence of 44.6%, cataracts at 19.4%, macular degeneration at 16.2%, and glaucoma at 2.3%, with significant differences across ages and genders. A potential link was found between myopia and an increased risk of cataracts and macular degeneration, but not with glaucoma. Additionally, a higher dietary intake of carbohydrates, polyunsaturated and n-6 fatty acids, vitamins, and minerals correlated with lower risks of these diseases, underscoring the importance of the diet in managing and preventing age-related eye conditions. These findings highlight the need for dietary considerations in public health strategies and confirm myopia as a significant risk factor for specific eye diseases in the aging Korean population.

## 1. Introduction

In recent decades, the life expectancy of the global population has risen significantly, leading to a proportional increase in the elderly demographic. This trend is notably pronounced in Korean society, which is aging at an accelerated rate. Data from the Korea National Statistical Office (2022) indicate that individuals aged 65 and older constitute 17.5% of Korea’s total population, a figure projected to surpass 20% by 2025 [1]. Moreover, the 2021 figures from the same source reveal that the average life expectancy for Koreans stands at 83.6 years—with men at 80.6 years and women at 86.6 years—registering an average annual increment of 0.4 years [2].

These demographic shifts are transforming public health into a central area of concern, encompassing socio-economic aspects as well as age-related health issues. Of particular concern among age-associated ailments is geriatric eye disease, which poses a significant threat to vision and, consequently, to the overall quality of life—more so than the deterioration of other bodily functions. The World Health Organization (WHO) reports that the leading causes of visual impairment and blindness globally are refractive errors, cataracts, glaucoma, and macular degeneration [3]. It is of note that in developed nations, age-related macular degeneration, along with glaucoma and diabetic retinopathy, are reported as the primary causes of blindness [3].

Three principal ocular diseases are predominantly associated with blindness in the elderly: cataracts, glaucoma, and age-related macular degeneration. Cataracts, a leading cause of blindness globally, manifest as a clouding of the eye’s lens, leading to progressive visual impairment [4]. Age-related macular degeneration, on the other hand, is a complex, multifactorial disease characterized by deterioration within the macula—the central area of the retina—resulting in a gradual loss of central vision and generally carrying a poor prognosis [5]. Glaucoma is yet another progressive condition that damages the optic nerve, often culminating in irreversible vision loss [6]. Furthermore, myopia—a refractive error where distant objects appear blurred due to the elongation of the eyeball—is seeing a rise in prevalence worldwide, with a particular increase in Asian populations, including that of Korea [7,8,9]. Historically, myopia has been primarily regarded as an isolated visual impairment predominantly affecting younger demographics. Nevertheless, recent findings suggest that myopia may be a contributory risk factor for various ocular diseases in later life [10,11]. In Korea, similar to other nations, the prevalence of geriatric eye diseases is rising in tandem with the aging population trend [12]. This increase in chronic eye conditions among the elderly is anticipated to pose a significant national burden, particularly in the context of social issues [13]. To effectively address these age-related eye diseases, it is crucial to adopt a dual approach that encompasses both clinical and public health strategies as well as the management of nutritional intake through diet. This holistic strategy should be recognized and implemented at both the national health and public health levels.

Thus, in light of the growing prevalence of myopia and its potential implications for public health, this study investigates the relationship between myopia and major age-related eye diseases such as cataracts, glaucoma, and macular degeneration in Koreans aged over 40. These diseases were selected due to their significant impact on vision and quality of life in the aging population. The association between myopia and these conditions is of particular interest, as it could offer insights into potential preventive and management strategies for age-related eye diseases. Examining the relationship between these eye diseases and nutrients that can be obtained through the diet is also considered a significant aspect of this study. This study aims to contribute to the understanding of these relationships using the comprehensive data available from the 7th Korea National Health and Nutrition Examination Survey (KNHANES VII, 2018) survey, enriching the discourse on the intersection of age-related ocular health.

## 2. Materials and Methods

### 2.1. Data Resource and Study Population

**Figure 1:**
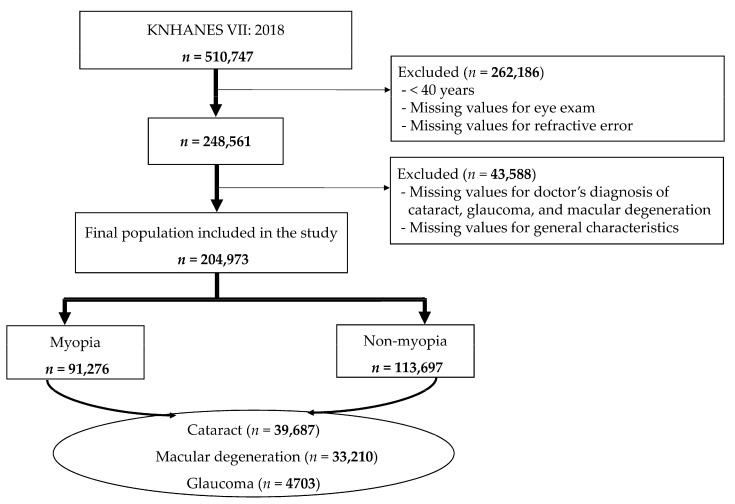
Participant selection flowchart.

This investigation focused on individuals aged 40 years and older within the 510,747 participants of the 7th KNHANES, conducted by the Korea Centers for Disease Control and Prevention. The initial dataset underwent a screening process to exclude participants who did not undergo an eye examination or lacked data pertaining to refractive errors. Furthermore, individuals missing data on cataracts, glaucoma, and macular degeneration—which were the conditions under study—as well as those missing general variable information were also excluded. After this filtering process, the study sample was consolidated to 204,973 individuals. These were categorized into two groups for analysis: those with myopia and those without myopia (Figure 1).

In this study, we focused our analysis on individuals aged 40 years and above. This decision was based on the established understanding that the prevalence and risk of age-related eye diseases such as cataracts, glaucoma, and macular degeneration significantly increase with age.

The KNHANES VII 2018 protocol was reviewed and approved by the Research Ethics Committee of the Korea Centers for Disease Control and Prevention (IRB number: 2018-01-03-P-A). All procedures were conducted in strict accordance with the principles outlined in the Declaration of Helsinki.

### 2.2. Demographic and Socio-Economic Data

The KNHANES methodically selects 25 households from 192 regions each year, using a probability sampling technique. This approach ensures a diverse and representative sample, encompassing approximately 10,000 individuals aged one year and above. The survey is designed to cater to the specific life cycle characteristics of each participant, thereby applying tailored survey items accordingly.

The survey process is conducted over a span of three days in each designated survey district. During this period, a mobile examination vehicle is dispatched to the area to facilitate both health checkups and surveys. The duration for each adult participant ranges from 1 h and 30 min to 2 h. The initial step involves verifying the identity of the participant, followed by the completion of a consent form, after which the survey proceeds.

The health checkup encompasses a variety of examination items, primarily focusing on the detection of chronic diseases such as obesity, hypertension, diabetes, and dyslipidemia. This is achieved through measurements of height and weight, blood pressure checks, as well as blood and urine tests, including oral examinations. In parallel, the health survey delves into aspects of health behavior, such as smoking habits, alcohol consumption, physical activity, and mental health. These are assessed through comprehensive questionnaires, ensuring a thorough understanding of the participant’s health-related lifestyle and behaviors.

Socio-demographic and socio-economic variables were gathered through interviewer-administered surveys. Health behavior data, including smoking and alcohol consumption habits, were collected using self-administered questionnaires. Body mass index (BMI) was calculated using the formula weight in kilograms divided by the square of height in meters (kg/m^2^). Current smoking status was defined as an individual who has smoked over 100 cigarettes in their lifetime and who currently smokes. Heavy alcohol consumption was characterized as consuming seven or more drinks per occasion for men, and five or more drinks for women, with this level of consumption occurring more than twice a week. Physical activity was defined as engaging in at least 30 min of walking, more than five days a week, during the preceding week. Additional variables included in the analysis were the participant’s education level, household income, hours spent on daily short-distance work, and the period since their last eye examination. For the purpose of this analysis, participant residences were classified as urban or rural.

### 2.3. Ocular Examinations

#### 2.3.1. Myopia Criteria

The measurement of refractive error was performed using an automatic refractometer (KR-8800, Topcon, Tokyo, Japan). Ocular examinations, including refractive error measurements, were conducted at the survey sites of the KNHANES, which are equipped with the necessary ophthalmologic instruments. Refractive errors were quantified by the spherical equivalent (SE); myopia was defined as SE ≤ −0.50 diopters (D). The diagnosis of eye diseases, including cataracts, glaucoma, and macular degeneration, was ascertained through interview surveys that relied on previously documented physician diagnoses.

#### 2.3.2. Cataract

Slit lamp examination (Haag-Streit BQ-900, Haag-Streit AG, Koeniz, Switzerland) was performed for the determination of cataract by ophthalmologists. Cataract was graded according to the Lens Opacities Classification System III [14] and was defined as a diagnosis of nuclear, cortical, or subcapsular cataract in at least one eye [15].

#### 2.3.3. Age-Related Macular Degeneration

Fundus photographs were taken with a digital non-mydriatic fundus camera (TRC-NW6S, Topcon) and a Nikon D-80 digital camera (Nikon, Tokyo, Japan). Fundus images were graded for preliminary and detailed grading in accordance with the International Age-related Maculopathy Epidemiological Study Group grading protocol [16]. In this study, we did not distinguish between early and late AMD, but included both in the prevalence of macular degeneration.

#### 2.3.4. Glaucoma

Intraocular pressure (IOP) was measured with a Goldmann tonometer attached to a slit lamp. Frequency doubling perimetry (FDT) was performed in participants who met the criteria for suspected glaucoma. The FDT (Humphrey Matrix, Carl Zeiss Meditec Inc., Dublin, CA, USA) test with the N-30-1 screening program was used for participants with IOP elevation ≥22 mm Hg or glaucomatous optic disc. To diagnose glaucomatous structural damage, optical coherence tomography (Stratus OCT, Carl Zeiss Meditec, Inc., Dublin, CA, USA) was used to observe the thickness of the retinal nerve fiber layer around the optic disc. The ophthalmologist’s criteria for glaucoma were based on the International Society for Geographic and Epidemiological Ophthalmology, and glaucomatous structural damage and glaucomatous visual field damage were considered category I or II or III [17].

The Epidemiologic Survey Committee of the Korean Ophthalmologic Society reviewed the quality of the ophthalmic survey [15,18]. To analyze trends in the prevalence of myopia and other eye diseases, the study population was stratified into five age cohorts: 40–49 years, 50–59 years, 60–69 years, 70–79 years, and 80 years or older.

### 2.4. Dietary Intake Assessment

The KNHANES nutritional survey captures data on dietary habits, food frequency, and actual consumption through direct interviews [19]. In our analysis, nutrient intake data were sourced from this dataset, which includes details on 24 different nutrients such as energy, protein, fats, and carbohydrates. Dietary data were collected using a 24 h recall method, allowing participants to provide detailed accounts of all meals and food items consumed within a single day. To evaluate the adequacy or excess of nutrient intake relative to age and gender, we referenced the 2020 Dietary Reference Intakes for Koreans [20].

### 2.5. Statistical Analysis

Data were statistically processed using the SAS software (version 9.4; SAS Institute Inc., Cary, NC, USA). Analysis was conducted by applying a complex sample design method, accounting for cluster variables, stratification variables, and the weights as outlined in the 7th National Health and Nutrition Examination Survey. Descriptive statistics were reported as mean ± standard deviation (SD) for continuous variables and as frequencies for categorical variables. The chi-square test was used to compare categorical data between myopic and non-myopic groups. For continuous variables, comparisons between two groups were performed using analysis of covariance (ANCOVA). The adjusted odds ratio (OR) and 95% confidence intervals (CI) were calculated using multivariate logistic regression analysis. Two models were employed in the multivariate logistic regression analysis: Model 1 adjusted for age, gender, BMI, near work activity, education level, and household income; Model 2 included adjustments for age, gender, BMI, smoking status, heavy drinking, residential area, and physical activity. A *p*-value of <0.05 was considered to denote statistical significance in all analyses.

## 3. Results

### 3.1. Study Population and Characteristics

This study included 204,973 adults aged over 40 years, with a gender distribution of 44% men and 56% women. The mean age of participants was 58.70 ± 10.75 years, and the prevalence of myopia was 44.6%. The prevalence of myopia was higher among women (59.8%) compared to men (40.2%). The body mass index (BMI) was marginally higher in the non-myopia group than in the myopia group. The current smoking rates were 15.7% for the myopic group and 14.5% for the non-myopic group. Rates of excessive alcohol consumption were comparable between the myopic (10.9%) and non-myopic groups (10.6%).

Time spent on near work activities exceeding two hours was reported by 43.2% in the myopia group, compared to 23.9% in the non-myopia group. Engagement in physical activity was higher in the myopia group, with 41.3% being active, against 38.5% in the non-myopia group. Higher levels of education and household income were observed in the myopic group, with 76.3% and 62.1%, respectively, in contrast to 52.8% and 51.9% in the non-myopic group. A larger proportion of myopic individuals (71.1%) resided in urban areas compared to the non-myopic group (66.9%).

Ophthalmological examinations within the past year were reported by 37% of participants in both groups. However, about 25% of each group had never undergone an ophthalmological examination (Table 1).

### 3.2. Prevalence of Myopia and Non-Myopia by Age

In this study, the overall prevalence of myopia was found to be 44.6%, with high myopia constituting 3.6% of this figure. Age-specific analysis revealed that myopia prevalence was markedly high at 74.9% among participants in their 40s. This prevalence declined to 45.1% in the age group of participants in their 50s. While there was a decreasing trend observed with advancing age, the prevalence of myopia remained substantial, with more than a quarter (25%) of participants in their 60s and above being myopic (Table 2).

### 3.3. Prevalence of Eye Diseases

**Figure 2:**
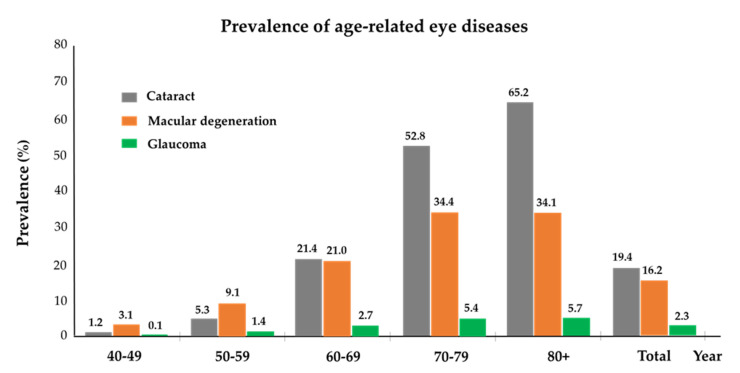
Age-wise distribution of the prevalence of cataracts, macular degeneration, and glaucoma in Korean adults aged 40 years and older.

The prevalence of cataracts, macular degeneration, and glaucoma among adults aged 40 and over is detailed in Table 3 and illustrated in Figure 2, delineated by age and gender. Overall, the prevalence of cataracts was 19.4%, with a higher incidence in women (21.7%) compared to men (16.3%). In the 50s age group, the prevalence was 5.3%, but this figure rose markedly to 21.4% in the 60s age group, and further escalated to over 55.2% in those aged 70 and above, illustrating a clear age-related increase.

For macular degeneration, the overall prevalence was noted at 16.2%, with a higher frequency in men (17.6%) than in women (15.1%). The condition was present in 9.1% of individuals in their 50s, climbed to 21.0% in the 60s age bracket, and peaked at 34.3% in those aged 70 and older, confirming that the likelihood of macular degeneration rising with age.

Glaucoma presented with an overall prevalence of 2.3%, occurring more frequently in women (2.5%) than in men (2.1%). The prevalence increased from 2.7% in those in their 60s to 5.4% in the 70-plus demographic, indicating a progression in prevalence with advancing age.

The data revealed significant differences in the prevalence of cataracts, macular degeneration, and glaucoma when analyzed against both age and gender, with these differences being statistically significant (all, *p* < 0.0001).

### 3.4. Association between Age-Related Eye Disease and Myopia

This study examined the association between age-related eye diseases, such as cataracts, glaucoma, and macular degeneration, and myopia. Two statistical models were employed to adjust for confounding variables: Model 1 incorporated age, gender, BMI, near work activity, education, and household income, while Model 2 included smoking, heavy drinking, residential area, and physical activity as covariates.

The analysis of both Model 1 and Model 2 revealed a significant association between myopia in individuals over 40 and an increased risk of cataracts with advancing age. Specifically, in Model 1, the odds ratios (ORs) for cataracts across different age groups with myopia were as follows: in the 40s group, OR = 0.007 (95% CI, 0.007–0.009); in the 50s group, OR = 0.025 (95% CI, 0.023–0.028); in the 60s group, OR = 0.135 (95% CI, 0.123–0.147); and in the 70s group, OR = 0.858 (95% CI, 0.787–0.936) (all, *p* < 0.0001). Model 2 showed similar trends, with the ORs for cataracts being as follows: in the 40s group, OR = 0.004 (95% CI, 0.004–0.005); in the 50s group, OR = 0.015 (95% CI, 0.014–0.017); in the 60s group, OR = 0.113 (95% CI, 0.103–0.123); and in the 70s group, OR = 0.865 (95% CI, 0.794–0.943) (all, *p* < 0.0001).

Regarding macular degeneration, Model 1 indicated increasing ORs with age: in the 40s group, OR = 0.067 (95% CI, 0.061–0.075); in the 50s group, OR = 0.148 (95% CI, 0.135–0.162); in the 60s group, OR = 0.402 (95% CI, 0.369–0.438); and in the 70s group, OR = 0.728 (95% CI, 0.671–0.790) (all, *p* < 0.0001). Model 2 also demonstrated a progressive increase in ORs with age: in the 40s group, OR = 0.050 (95% CI, 0.045–0.055); in the 50s group, OR = 0.148 (95% CI, 0.136–0.161); in the 60s group, OR = 0.452 (95% CI, 0.417–0.491); and in the 70s group, OR = 0.786 (95% CI, 0.724–0.852) (all, *p* < 0.0001). However, this study found no significant association between the prevalence of glaucoma and myopia across age groups (Table 4).

### 3.5. Nutrient Intake in Relation to Age-Related Eye Diseases

In Table 5, the average daily nutrient intake is compared among participants over 40 years old with age-related eye diseases such as cataracts, glaucoma, and macular degeneration. It was found that individuals with cataracts consumed less energy but had a higher intake of protein, fat, especially saturated fatty acids, calcium, phosphorus, retinol, and riboflavin, as well as a lower intake of fiber and sugars. Those with glaucoma showed a lower overall energy consumption and carbohydrate intake but had higher protein, fat, and saturated and monounsaturated fatty acid intakes, as well as an increased intake of vitamin A, retinol, thiamin and riboflavin. Participants with macular degeneration had a similar energy intake compared to those without, but displayed distinct patterns in micronutrient intake, including higher retinol and vitamin C, but lower levels of calcium, phosphorus, niacin, and folate.

### 3.6. Nutritional Intake and Its Protective Role against Age-Related Eye Diseases

**Figure 3:**
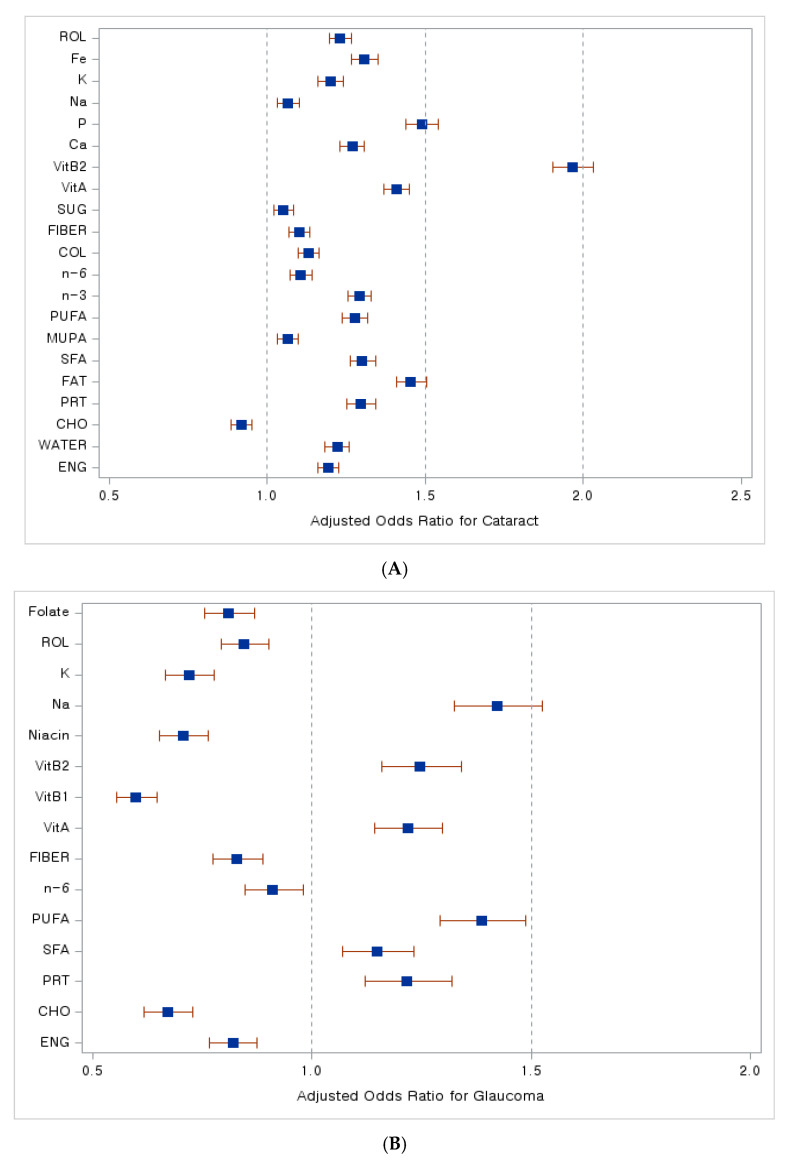

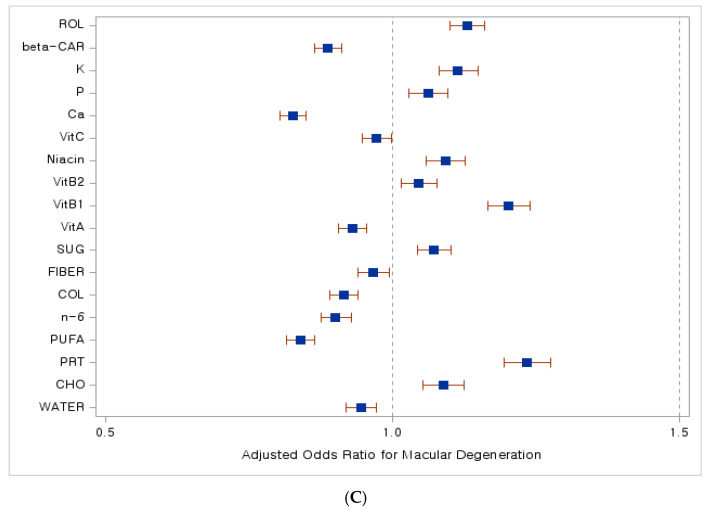
Dietary nutrient intake and its association with the risk of age-related eye diseases. A forest plot. (**A**) Adjusted odds ratio with cataract, (**B**) adjusted odds ratio with glaucoma, (**C**) adjusted odds ratio with macular degeneration. Abbreviations: ENG, energy; CHO, carbohydrates; PRT, proteins; SFA, saturated fatty acids; MUFA, monounsaturated fatty acids; PUFA, polyunsaturated fatty acids; n-3, Omega-3 fatty acids; n-6, Omega-6 fatty acids; COL, cholesterol; SUG, sugar; VitA, vitamin A; VitB1, vitamin B1; VitB2, vitamin B2; Ca, calcium; P, phosphate; Na, sodium; K, potassium; Fe, iron; beta-CAR, beta-carotene; ROL, retinol. Blue squares indicate adjusted odds ratio (OR) values, while red lines represent 95% confidence interval (CI) values.

In Figure 3, we present compelling forest plots that illustrate the relationship between dietary nutrient intake and the risk of various age-related eye diseases. In Figure 3A, we observe that a higher intake of carbohydrates is associated with a lower risk of developing cataracts, as indicated by an adjusted odds ratio (aOR) less than 1. Moving to Figure 3B, the data reveal that increased consumption of energy, carbohydrates, n-6 fatty acids, dietary fiber, thiamine, niacin, potassium, retinol, and folate corresponds to a reduced risk of glaucoma, again reflected in aORs less than 1. Lastly, Figure 3C highlights the relationship with macular degeneration, where an increased intake of water, polyunsaturated and n-6 fatty acids, cholesterol, dietary fiber, vitamins A and C, calcium, and beta-carotene is linked to a lower risk of this disease, as evidenced by aORs below 1.

## 4. Discussion

The major finding of this study highlighted a significant association between myopia and age-related eye diseases, specifically cataracts and macular degeneration, among Korean adults aged 40 and above. This underscores the public health challenge of managing vision impairments in an aging population. The absence of a similar link between myopia and glaucoma warrants further investigation. These insights not only emphasize the need for targeted strategies to manage vision impairments associated with aging but also the potential implications of myopia in the development of certain age-related eye conditions. Furthermore, the significant correlations found between dietary nutrient intake and these eye diseases emphasize the protective role of a nutrient-rich diet. This study highlighted that a higher intake of certain nutrients—carbohydrates, polyunsaturated and n-6 fatty acids, vitamins, and minerals—was associated with a lower risk of developing cataracts, glaucoma, and macular degeneration, suggesting the importance of the diet in the management and prevention of these age-related eye diseases. This study enriches the discourse on the intersection of age-related ocular health by providing comparative insights into how these associations are observed both locally within the Korean demographic and in broader international contexts, reflecting both unique and common trends in age-related ocular health management.

Our findings revealed a clear hierarchy in the prevalence of these conditions, with cataracts being the most prevalent, followed by macular degeneration, and then glaucoma. This prevalence notably increased with advancing age. Specifically, the prevalence of cataracts in the Korean population aged 40 or older was found to be 19.7%, which escalated to 55.2% among those 70 or older. This trend of cataracts being the predominant form of visual impairment in older adults aligns with global observations, where cataracts are recognized as a leading age-related ophthalmic condition and a significant public health concern [21,22]. The development of cataracts is understood to be a complex interplay of factors, including oxidative stress, genetic predispositions, and environmental influences such as ultraviolet radiation and dietary patterns, which may be particularly relevant in our study cohort [23,24,25]. In this study, we explored the prevalence of age-related eye diseases among Koreans aged 40 and above, which was based on the established understanding that the prevalence and risk of age-related eye diseases significantly increase with age [12,26,27,28,29,30]. Including younger participants, especially those under 40 years, could have led to an underestimation of these diseases’ actual prevalence. Our methodology aligns with standard practices in epidemiological research on age-related eye diseases, where focusing on an older population ensures a more accurate representation of the prevalence and associations of these conditions. This approach also allows for a more relevant assessment of the relationship between myopia and these age-related diseases.

This study’s results also draw attention to gender differences in the risk of cataract development, suggesting the potential role of biological factors such as estrogen. Understanding these differences is vital for developing effective preventive measures. Our research indicates that the risk of cataract development was higher in women than in men. This differential risk is posited to be linked to the protective role of estrogen, an antioxidant, against cataract formation. Postmenopausal reductions in estrogen are suggested to increase cataract risk [31]. While it has been suggested that diminished estrogen levels might also heighten the risk of macular degeneration in women [32], our study interestingly found a slightly higher prevalence of this condition in men. Zetterberg has highlighted the necessity of considering both biological and socio-economic factors to fully understand the gender disparities observed in the prevalence of age-related eye diseases [31].

Xu et al.’s study on vision loss in Chinese adults and the elderly highlights cataracts and degenerative myopia as leading causes, mirroring trends observed in Western countries, though with notable differences in the prevalence of age-related macular degeneration and diabetic retinopathy [33]. The WHO’s reports [3] and Wong et al.’s meta-analysis [34] underscore the varying prevalence of macular degeneration globally, with our study revealing a notably high prevalence among Koreans aged 40 or older, especially after 60, posing a significant public health challenge. Globally, macular degeneration is responsible for 7–8% of blindness and most commonly affects those over the age of 60 [30,35]. While the precise etiology of macular degeneration remains elusive, it is widely accepted that both genetic predispositions and various environmental risk factors contribute to its onset. Genetic influence on susceptibility to macular degeneration is well-documented, with numerous genetic variants identified as consistent risk factors [5,36]. Additionally, individual risk factors such as age, smoking, diet, family history, and cardiovascular health have been implicated in increasing the likelihood and accelerating the progression of macular degeneration [37,38].

Our findings on glaucoma, a major cause of irreversible blindness, align with global statistics, showing a lower prevalence in the Korean population compared to cataracts and macular degeneration, but with an increase in older age groups. Glaucoma, often referred to as the ‘silent thief of sight,’ is a condition characterized by optic nerve damage, frequently attributable to elevated intraocular pressure that compromises the blood supply to the optic nerve [39]. The disease insidiously narrows the field of vision, typically beginning at the periphery, and can progress unnoticed until it reaches an advanced stage. Globally recognized as a leading cause of irreversible blindness, glaucoma’s major risk factors include aging, family history, high myopia, hypertension, and diabetes, all of which elevate the risk of its development [40,41,42]. Tham et al.’s meta-analysis, which evaluated the prevalence of glaucoma in individuals aged 40 to 80, revealed a global prevalence of 3.5%, with the highest rates observed in Africa, at 4.8%, and Asia, at 3.4% [43]. Our study noted that in Korea, the prevalence of glaucoma in individuals aged 40 and above was 2.3%, which is lower compared to the prevalence of cataracts and macular degeneration. Notably, the prevalence among those in their 70s or older was recorded at 5.4%.

Historically, cataracts were the primary cause of blindness in Korea 20 to 30 years ago. Today, cataract surgery, with its high success rate, is the most commonly performed surgical procedure worldwide, including in Korea [44,45]. Advances in surgical technology have evolved to a point where postoperative vision can rival that of a younger individual [46]. Korea’s implementation of a national health insurance system and the introduction of a comprehensive fee system (diagnosis-related group) for cataract surgery, coupled with the actual cost insurance system, has considerably lowered the economic barriers to surgical intervention [47]. However, patient awareness, accessibility of medical facilities, and socio-economic status still play a significant role in the treatment of cataracts [48]. Macular degeneration and glaucoma, two of the three principal causes of blindness, are on the rise among the adult population in Korea. Data from the Health Insurance Review and Assessment Service indicate a dramatic surge in the prevalence of these conditions. Between 2010 and 2015, cases of macular degeneration increased by 104.8%, with a marked increase of 167.7% observed in individuals aged 70 and above. Concurrently, glaucoma prevalence increased by 99.0% across all age groups, and by 147.1% in those in their 70s or older. Notably, macular degeneration has the fastest-growing rate of incidence and has become the leading cause of blindness in the elderly [49]. As cataract surgery becomes increasingly accessible and effective in Korea, the focus shifts to the rising prevalence of macular degeneration and glaucoma, highlighting the need for enhanced public awareness and early detection strategies to combat these growing public health concerns.

Despite the rising prevalence of these eye diseases, largely attributed to an aging population, our findings highlight a critical gap in the awareness and management of age-related eye diseases among the Korean population, emphasizing the urgent need for enhanced public health measures and early detection strategies. Only 3.5% of surveyed Korean adults over 40 were aware of having macular degeneration, and 25.8% were aware of having glaucoma. The treatment rates were equally low, with 1.4% for macular degeneration and 20.3% for glaucoma. Among diabetic individuals, a mere 23.5% had undergone a fundus examination to screen for ocular complications [50]. These statistics underscore the need for greater public health initiatives, such as the Korean Academy of Ophthalmology’s recommendation to include fundus photography in national health screenings to detect macular degeneration and glaucoma earlier. The insidious nature of age-related macular degeneration and glaucoma, which often present with no discernible symptoms until they have significantly advanced, complicates early detection. Moreover, when these diseases afflict only one eye, changes in vision may go unnoticed as patients depend on their unaffected eye. Unlike cataracts, which are treatable with surgery, vision loss from glaucoma and macular degeneration is irreversible. Typically diagnosed in individuals in their 50s or 60s, and with the average life expectancy in Korea around 84 years, there is a high potential for a significant portion of the population to experience blindness within their lifetimes. Therefore, incorporating screenings for glaucoma and macular degeneration into national health examinations is imperative. Early detection and timely intervention through regular eye examinations may be the most effective strategy to mitigate the risk of blindness from these diseases.

The escalating prevalence of myopia globally, and specifically In the Korean population aged 40 and above, underscores the need for heightened attention to this condition, given its potential to cause significant vision impairment and blindness. Myopia has been on a rapid rise for decades, with projections suggesting that by 2050, nearly half (49.8%) of the global population will be affected, and high myopia will comprise 9.8% of this demographic [7]. Tideman et al. have forecasted a 7- to 13-fold increase in visual impairment within high myopia risk groups by 2055 [51]. Our study reflects a similar trend in the Korean population aged 40 and above, showing a myopia prevalence of 44.6%, with high myopia accounting for 3.6%. There is a continuous upward trajectory in these numbers. Although often disregarded because of its correctability through glasses, contact lenses, or refractive surgery, myopia should not be overlooked due to its potential to cause blindness. High myopia, in particular, significantly heightens the risk of developing vision-impairing conditions such as myopic macular degeneration, cataracts, retinal detachment, and glaucoma, with the risk escalating in tandem with the severity of myopia [52,53]. Furthermore, even low degrees of myopia pose safety concerns due to an elevated risk of retinal detachment and other eye comorbidities [10,54]. The gravest concern is myopic macular degeneration, which leads to irreversible vision loss and, unlike other eye conditions, lacks effective treatment options [55,56]. Considering that myopia begins in childhood and its sight-threatening complications manifest later, early intervention may be crucial in preventing serious vision-threatening eye diseases in Korea’s aging population.

As we explore the associations found in our study between myopia, dietary nutrients, and age-related eye diseases, it is crucial to contextualize these findings within the wider body of existing research. Several studies have investigated these relationships, highlighting both genetic and environmental factors influencing ocular health. Previous studies provide insights into the complex dynamics of myopia and its correlation with other eye conditions [57,58]. Furthermore, the role of dietary nutrients in eye health has been substantiated by researchers who emphasize the protective effects of carotenoids and antioxidants against macular degeneration and cataracts [59,60]. Another study also extends this discussion to the sensory benefits of omega-3 fatty acids [61]. These studies form a vital backdrop against which our findings can be assessed, suggesting that dietary interventions could significantly contribute to the management and prevention of age-related eye diseases linked with myopia. The correlation between higher intake of specific nutrients and a reduced risk of such conditions aligns with our observations, reinforcing the potential of nutritional strategies in ocular health protocols (Table 6 and Table 7).

Following the analysis of nutrient intake in relation to age-related eye diseases as presented in this study, it is pertinent to consider specific nutrients known for their significance in eye health. Lutein and zeaxanthin, vital carotenoids for ocular health, are found in leafy green vegetables such as kale, spinach, broccoli, and lettuce, as well as in egg yolks, making them the primary dietary sources of xanthophylls [75]. These carotenoids are crucial as they accumulate in the macula, the most sensitive part of the retina, aiding in clear central vision. Our body cannot synthesize these carotenoids; hence, their dietary or supplemental intake is essential. Research indicates that lutein may play a protective role against age-related eye diseases like macular degeneration and cataracts [76,77]. Furthermore, zinc and vitamin A, present in red meat, dairy, legumes, and carrots, are fundamental for eye health. Zinc facilitates the transport of vitamin A from the liver to the eyes, contributing to melanin production—a pigment vital for eye protection from UV rays [78]. Similarly, vitamins C and E, found in foods like oranges, broccoli, nuts, and oils, are essential for maintaining eye structure and function. Vitamin C aids in collagen production, crucial for eye structure and increasingly important as collagen levels decline with age [79]. Vitamin E, a fat-soluble antioxidant, enhances the protective abilities of lutein, safeguarding retinal health [80]. This emphasis on specific nutrients and their dietary sources underlines the significance of diet in managing and preventing age-related eye conditions, a critical aspect often overlooked in public health discussions.

### 4.1. Clinical Implications

The clinical implications of our study suggest a nuanced approach to integrating dietary and lifestyle adjustments for managing and preventing age-related eye diseases associated with myopia. To enhance the practical relevance of our findings in the clinical setting, this section outlines specific applications derived from our study that can directly benefit clinical practice. Based on the observed correlations between myopia and age-related eye diseases such as cataracts and macular degeneration, we recommend that clinicians consider integrating early screening protocols for these conditions in patients with myopia. Early identification through routine examinations can lead to timely interventions, potentially slowing disease progression and improving patient outcomes. Additionally, our findings suggest that dietary modifications, particularly increased intake of nutrients shown to have protective effects against ocular deterioration, should be encouraged as part of standard clinical advice. Clinicians can guide patients on incorporating foods rich in antioxidants and anti-inflammatory properties, which may help mitigate the risk of cataracts and macular degeneration. Implementing these strategies not only helps in managing myopia but also addresses associated risks, offering a comprehensive approach to patient care in ophthalmology.

Furthermore, advancing our understanding of the complex interactions between diet and eye health will require collaborative efforts that not only bridge the gap between epidemiological data and clinical practice but also emphasize the development of large-scale, longitudinal studies that can provide more conclusive evidence and thus more robust guidelines. While this research study identifies potential correlations between nutrient intake and eye health, it is prudent to consider these findings preliminary. Clinicians should be cautious in translating these associations into direct nutritional recommendations without further evidence. Instead, these insights can guide more comprehensive assessments of dietary patterns in patients at risk for or currently managing eye diseases, potentially leading to personalized advice that supports overall ocular health [81]. This approach avoids definitive dietary prescriptions while still acknowledging the importance of nutrition in eye disease prevention and management.

### 4.2. Strengths and Limitations

The strengths of this study are highlighted by its use of comprehensive data from the 7th Korea National Health and Nutrition Examination Survey (KNHANES VII, 2018), with a robust sample size of 204,973 adults over 40. This large dataset underpins the investigation into the relationships between myopia, dietary nutrients, and age-related eye diseases, enhancing the reliability of findings. Detailed nutrient analysis provides insights into potential preventative dietary strategies, and adjusted multivariate logistic regression solidifies the credibility of the results. These attributes strengthen this study’s public health implications, emphasizing the importance of dietary management in eye disease prevention. However, despite these strengths, there are limitations that must be acknowledged in the subsequent section. These include potential biases inherent in self-reported data, the cross-sectional nature of this study, which limits causal inference, and the challenges associated with generalizing findings across different populations or ethnic groups. These limitations are essential for understanding the scope and applicability of this study’s conclusions in broader clinical and public health contexts.

Consequently, this study’s cross-sectional design restricts our ability to infer causal relationships and understand the mechanisms between myopia and age-related eye diseases. We focused on cataracts, glaucoma, and macular degeneration, which, while significant, limits our insight into the severity and progression of these conditions. Additionally, the absence of detailed treatment or surgical timelines in our data is a notable limitation. Another important limitation of our study lies in the classification and diagnosis of age-related eye diseases, particularly given the evolving nature of diagnostic equipment and criteria during the study period (2017–2021), in collaboration with the Korean Academy of Ophthalmology. While our classification of cataracts followed the Lens Opacities Classification System III and the assessment of fundus images was based on the International Age-related Maculopathy Epidemiological Study Group protocol, with glaucoma diagnosis aligning with criteria from the International Society for Geographic and Epidemiological Ophthalmology, there remains a question regarding the consistency of these classifications with real-world scenarios as defined by ICD-10 diagnostic codes. This discrepancy could influence the generalizability of our findings. This study acknowledges the need for further validation of these diagnostic methods, possibly through comparisons with self-reports, telephone-based surveys, or medical chart reviews, to enhance the reliability and applicability of our results in broader clinical and public health contexts. In addition, our method of nutrient evaluation, which relied on the 24 h recall technique, has inherent limitations in fully elucidating the relationship between eye diseases and nutrient intake. Twenty-four-hour recall depends on participants’ memory and honesty and may not reflect usual intake or account for day-to-day variations in the diet. Therefore, the precision of nutrient intake assessment might have been compromised, potentially impacting the strength of the observed associations.

### 4.3. Future Research

In our investigation of the possible links between age-related eye diseases—such as cataracts, macular degeneration, glaucoma—and myopia, we observed a potential correlation between myopia and the prevalence of cataracts and macular degeneration. However, no association was found between the prevalence of glaucoma and myopia. Despite evidence suggesting that myopia may impact the development and progression of various eye diseases, analyses and meta-analyses utilizing population-based data to probe the connections between myopia and age-related eye conditions like cataracts, macular degeneration, and glaucoma have yielded inconclusive results [82,83]. These aspects underscore the necessity for further longitudinal studies to comprehensively understand the dynamics between myopia and these eye diseases, particularly in terms of disease progression and management strategies. These studies will be essential to unravel the complex dynamics of how dietary factors may influence the development and progression of eye diseases. This approach not only aims to clarify the direct effects of nutrients on eye health but also seeks to develop and refine management strategies for these conditions. Additionally, future studies should explore more robust dietary assessment tools to better understand the dietary factors contributing to age-related eye diseases.

## 5. Conclusions

In conclusion, our study highlights the importance of comprehensive eye health strategies, particularly for individuals with myopia, in addressing the risk of age-related vision loss. The observed associations between myopia and the prevalence of cataracts and macular degeneration emphasize the need for increased public awareness and early detection strategies in managing these conditions. Crucially, our research also reveals significant correlations between dietary nutrient intake and the risk of age-related eye diseases. A diet rich in carbohydrates, polyunsaturated and n-6 fatty acids, vitamins, and minerals showed a protective association against the development of cataracts, glaucoma, and macular degeneration. These findings underscore the role of nutritional considerations in eye health and highlight the potential of diet-based interventions in preventing and managing age-related eye conditions. As such, incorporating dietary guidance into public health campaigns for eye health could greatly benefit the aging population. Despite these valuable insights, further research is necessary to fully understand the complex interplay between myopia, diet, and age-related eye diseases. Advocating for comprehensive eye health care, especially among those with myopia, remains crucial to supporting overall ocular health and reducing the burden of vision loss in an aging society.

## Figures and Tables

**Table 1:** Participant characteristics by myopia status for individuals aged 40 years and older.

	Myopia N (%)	Non-Myopia N (%)	*p*-Value
Number	91,276 (44.6%)	113,697 (55.4%)	
Age (years)	54.88 ± 11.55 ^(3)^	62.51 ± 9.94	<0.0001 ^(1)^
Gender			<0.0001 ^(2)^
Male	36,704 (40.2%)	52,467 (46.2%)	
Female	54,572 (59.8%)	61,230 (53.8%)	
Refractive error (SE *) (D)	−2.38 ± 2.35	+0.78 ± 1.06	<0.0001
BMI ** (kg/m^2^)	23.91 ± 3.24	24.16 ± 3.08	<0.0001
Current smoking			<0.0001
Yes	14,328 (15.7%)	16,484 (14.5%)	
No	76,948 (84.3%)	97,213 (85.5%)	
Heavy alcohol			0.0253
Yes	9957 (10.9%)	12,053 (10.6%)	
No	81,319 (89.1%)	101,644 (89.4%)	
Near work activity			<0.0001
≤2 h/D	51,861 (56.8%)	86,473 (76.1%)	
>2 h/D	39,415 (43.2%)	27,224 (23.9%)	
Education			<0.0001
Less than high school	21,622 (23.7%)	53,715 (47.2%)	
High school and above	69,654 (76.3%)	59,982 (52.8%)	
Household income			<0.0001
Lowest quartile	13,938 (15.3%)	24,445 (21.5%)	
2nd quartile	20,754 (22.7%)	30,265 (26.6%)	
3rd quartile	27,210 (29.8%)	29,144 (25.6%)	
Highest quartile	29,374 (32.3%)	29,843 (26.3%)	
Residence			<0.0001
Urban	64,914 (71.1%)	76,089 (66.9%)	
Rural	26,362 (28.9%)	37,608 (33.1%)	
Physical activity			<0.0001
Yes	37,655 (41.3%)	43,792 (38.5%)	
No	53,621 (58.7%)	69,905 (61.5%)	
Last eye exam			<0.0001
<1 year	33,784 (37.0%)	43,299 (38.0%)	
1 year ≤ exam < 3 years	15,230 (16.7%)	18,414 (16.2%)	
≥3 years	18,346 (20.1%)	23,489 (20.7%)	
None	23,916 (26.2%)	28,495 (25.1%)	

^(1)^ Different between two groups at α = 0.05 by ANCOVA test. ^(2)^ Different between two groups at α = 0.05 by chi-square test. ^(3)^ Mean ± standard deviation (SD). * SE, spherical equivalent; ** BMI, body mass index.

**Table 2:** Age and gender distribution of myopia prevalence.

Age Group (Years)	Myopia N (%)	Non-Myopia N (%)	*p*-Value
40–49	38,333 (74.9%)	12,867 (25.1%)	<0.0001
50–59	25,021 (45.1%)	30,556 (54.9%)	<0.0001
60–69	14,113 (26.4%)	39,388 (73.6%)	<0.0001
70–79	10,323 (28.8%)	25,631 (71.2%)	<0.0001
80+	3486 (39.9%)	5255 (60.1%)	<0.0001
Total	91,276 (44.6%)	113,697 (55.4%)	<0.0001
Gender			
Male	36,704 (41.2%)	52,467 (58.8%)	<0.0001
Female	54,572 (47.1%)	61,230 (52.9%)	<0.0001

**Table 3:** Prevalence of cataracts, glaucoma, and age-related macular degeneration, stratified by age and gender.

Age (Years)	Cataract N (%)			Glaucoma N (%)			Macular Degeneration N (%)		
	Male	Female	*p*-Value	Male	Female	*p*-Value	Male	Female	*p*-Value
40–49	261 (1.2%)	360 (1.2%)	0.8841	0 (0.0%)	57 (0.2%)	<0.0001	903 (4.2%)	668 (2.2%)	<0.0001
50–59	875 (3.9%)	2079 (6.3%)	<0.0001	230 (1.0%)	573 (1.7%)	<0.0001	2346 (10.5%)	2726 (8.2%)	<0.0001
60–69	3393 (13.4%)	8047 (28.5%)	<0.0001	512 (2.0%)	907 (3.2%)	<0.0001	5006 (19.8%)	6217 (22.0%)	<0.0001
70–79	7613 (46.5%)	11,357 (58.0%)	<0.0001	857 (5.2%)	1069 (5.5%)	0.3660	6341 (38.8%)	6020 (30.7%)	<0.0001
80+	2412 (62.6%)	3290 (67.3%)	<0.0001	232 (6.0%)	266 (5.4%)	0.2481	1083 (28.1%)	1900 (38.9%)	<0.0001
Total	14,554 (16.3%)	25,133 (21.7%)	<0.0001	1831 (2.1%)	2872 (2.5%)	<0.0001	15,679 (17.6%)	17,531 (15.1%)	<0.0001
*p*-value	<0.0001	<0.0001		<0.0001	<0.0001		<0.0001	<0.0001	

**Table 4:** Odds ratios (95% confidence intervals) for the association between myopia and eye diseases across different age groups.

	Myopia					Non-Myopia				
	N	Model 1 ^(1)^ OR (95% CI)	*p*	Model 2 ^(2)^ OR (95% CI)	*p*	N	Model 1 OR (95% CI)	*p*	Model 2 OR (95% CI)	*p*
Cataract										
40–49	417	0.007 (0.007–0.009)	<0.0001	0.004 (0.004–0.005)	<0.0001	204	0.012 (0.010–0.014)	<0.0001	0.010 (0.009–0.012)	<0.0001
50–59	1047	0.025 (0.023–0.028)	<0.0001	0.015 (0.014–0.017)	<0.0001	1907	0.047 (0.043–0.051)	<0.0001	0.041 (0.038–0.044)	<0.0001
60–69	3078	0.135 (0.123–0.147)	<0.0001	0.113 (0.103–0.123)	<0.0001	8362	0.179 (0.168–0.191)	<0.0001	0.166 (0.156–0.176)	<0.0001
70–79	7089	0.858 (0.787–0.936)	<0.0001	0.865 (0.794–0.943)	<0.0001	11,881	0.545 (0.513–0.580)	<0.0001	0.553 (0.520–0.588)	<0.0001
80+	2507	1		1		3195	1		1	
Glaucoma										
40–49	0	-	-	-	-	57	0.244 (0.178–0.334)	<0.0001	0.098 (0.073–0.132)	<0.0001
50–59	377	0.158 (0.132–0.190)	0.9423	0.194 (0.165–0.228)	0.9396	426	0.645 (0.538–0.774)	0.6983	0.321 (0.271–0.382)	<0.0001
60–69	717	0.674 (0.579–0.785)	0.9056	0.713 (0.617–0.823)	0.9060	702	0.617 (0.525–0.726)	0.5920	0.452 (0.385–0.530)	0.3195
70–79	865	1.142 (0.991–1.315)	0.8923	1.116 (0.970–1.284)	0.8945	1061	1.040 (0.893–1.211)	<0.0001	1.057 (0.908–1.230)	<0.0001
80+	289	1		1		209	1		1	
MD										
40–49	1137	0.067 (0.061–0.075)	<0.0001	0.050 (0.045–0.055)	<0.0001	434	0.083 (0.074–0.094)	<0.0001	0.076 (0.068–0.086)	<0.0001
50–59	2037	0.148 (0.135–0.162)	<0.0001	0.148 (0.136–0.161)	<0.0001	3035	0.251 (0.233–0.271)	<0.0001	0.248 (0.231–0.266)	<0.0001
60–69	2816	0.402 (0.369–0.438)	<0.0001	0.452 (0.417–0.491)	<0.0001	8407	0.611 (0.573–0.652)	<0.0001	0.615 (0.577–0.656)	<0.0001
70–79	3240	0.728 (0.671–0.790)	<0.0001	0.786 (0.724–0.852)	<0.0001	9121	1.223 (1.147–1.304)	<0.0001	1.195 (1.120–1.274)	<0.0001
80+	1338	1		1		1645	1		1	

^(1)^ Adjusted OR for age, gender, BMI, near work, education, and household income. ^(2)^ Adjusted OR for age, gender, BMI, smoking, heavy drinking, residential area, and physical activity. Abbreviations: OR, odds ratio; CI, confidence interval; MD, macular degeneration.

**Table 5:** Average daily nutrient intake among participants over 40 with age-related eye diseases.

	2020 DRI ^(8)^		Cataract		Glaucoma		Macular Degeneration	
	Male	Female	No (N = 165,286)	Yes (N = 39,687)	No (N = 200,270)	Yes (N = 4703)	No (N = 171,763)	Yes (N = 33,210)
Energy (Kcal) ^(1)^	2200 ^(9)^	1700	1996.760 ± 1.885	1956.618 ± 4.219	1991.740 ± 1.657	1871.789 ± 10.902	*1989.619* * ± 1.806*	*1985.721 * *± 4.260*
Carbohydrates (g) ^(2)^	130	130	307.928 ± 0.179	301.423 ± 0.400	307.035 ± 0.157	291.072 ± 1.033	307.405 ± 0.171	302.856 ± 0.403
Proteins (g)	60	50	71.871 ± 0.051	73.361 ± 0.115	72.109 ± 0.045	74.303 ± 0.297	72.098 ± 0.049	72.477 ± 0.116
Fats (g)	-	-	40.484 ± 0.050	43.251 ± 0.111	40.963 ± 0.044	43.446 ± 0.287	*40.985 ± 0.048*	*41.201 ± 0.112*
SFAs (g) ^(3)^	-	-	12.307 ± 0.018	12.953 ± 0.041	12.423 ± 0.016	12.841 ± 0.107	12.413 ± 0.018	12.532 ± 0.042
MUSFAs (g) ^(4)^	-	-	12.948 ± 0.020	13.900 ± 0.045	13.113 ± 0.018	13.989 ± 0.117	*13.124 ± 0.019*	*13.178 ± 0.046*
PUSFAs (g) ^(5)^	-	-	11.542 ± 0.017	12.519 ± 0.038	11.701 ± 0.015	13.005 ± 0.098	*11.747 ± 0.016*	*11.646 ± 0.038*
n-6 fatty acids (g)	-	-	9.498 ± 0.014	10.133 ± 0.032	9.594 ± 0.013	10.785 ± 0.084	9.637 ± 0.014	9.538 ± 0.033
Cholesterol (mg)	300	300	230.826 ± 0.433	242.296 ± 0.970	*233.131* * ± 0.381*	*229.460 * *± 2.508*	235.187 ± 0.415	221.978 ± 0.979
Fiber (g)	30	20	28.536 ± 0.030	26.928 ± 0.067	28.255 ± 0.026	26.923 ± 0.174	28.297 ± 0.029	27.852 ± 0.068
Sugar (g)	-	-	58.318 ± 0.086	56.629 ± 0.193	*57.973 ± 0.076*	*58.760 ± 0.500*	58.134 ± 0.083	57.255 ± 0.195
Calcium (mg)	750	800	547.565 ± 0.735	565.694 ± 1.645	*550.986* * ± 0.646*	*554.853 * *± 4.253*	555.718 ± 0.704	527.061 ± 1.660
Phosphorus (mg)	700	700	1116.030 ± 0.731	1132.419 ± 1.637	*1119.052* * ± 0.643*	*1125.610 * *± 4.231*	1121.988 ± 0.700	1104.800 ± 1.652
Iron (mg)	10	8	13.121 ± 0.013	12.987 ± 0.030	*13.094* * ± 0.012*	*13.119 * *± 0.077*	*13.100* * ± 0.013*	*13.066 * *± 0.030*
Sodium (mg)	1500	1500	3569.355 ± 3.838	3511.706 ± 8.592	3555.653 ± 3.373	3666.356 ± 22.206	*3556.278* * ± 3.676*	*3568.098 * *± 8.672*
Potassium (mg)	3500	3500	3055.219 ± 2.359	3009.361 ± 5.282	3047.807 ± 2.074	2983.866 ± 13.652	3053.272 ± 2.260	3010.491 ± 5.331
Vitamin A (μg RAE ^(6)^)	750	600	402.217 ± 1.043	412.323 ± 2.335	403.305 ± 0.917	441.180 ± 6.035	400.838 ± 0.999	421.427 ± 2.356
β-carotene (μg)	-	-	*3199.480 * ^(10)^ * ± 6.351*	*3206.541 * *± 14.219*	*3201.555* * ± 5.583*	*3170.710 * *± 36.748*	3249.920 ± 6.077	2947.039 ± 14.338
Retinol (μg)	-	-	135.415 ± 0.875	144.931 ± 1.958	136.328 ± 0.769	176.853 ± 5.061	129.824 ± 0.837	175.703 ± 1.974
Thiamin (mg)	1.2	1.1	1.385 ± 0.001	1.397 ± 0.003	1.386 ± 0.001	1.443 ± 0.008	1.382 ± 0.001	1.414 ± 0.003
Riboflavin (mg)	1.5	1.2	1.591 ± 0.002	1.641 ± 0.003	1.598 ± 0.001	1.728 ± 0.009	1.602 ± 0.001	1.592 ± 0.003
Niacin (mg)	16	14	*13.431* * ± 0.012*	*13.443 * *± 0.026*	13.416 ± 0.010	14.170 ± 0.067	13.469 ± 0.011	13.249 ± 0.026
Folate (μg DFE ^(7)^)	400	400	*368.165* * ± 0.360*	*367.515 * *± 0.805*	368.330 ± 0.316	355.654 ± 2.081	370.084 ± 0.344	357.467 ± 0.812
Vitamin C (mg)	100	100	*69.419* * ± 0.194*	*69.065 * *± 0.435*	*69.326* * ± 0.171*	*70.376 * *± 1.125*	68.874 ± 0.186	71.811 ± 0.439

^(1)^ Age, sex, BMI, and energy (except energy)-adjusted least squares means (LSmeans); ^(2)^ Different between two groups at α = 0.05 by ANCOVA test adjusted for age, sex, BMI, and energy (except energy); ^(3)^ SFAs, saturated fatty acids; ^(4)^ MUSFAs, monounsaturated fatty acids; ^(5)^ PUSFAs, polyunsaturated fatty acids; ^(6)^ RAE, retinol activity equivalent; ^(7)^ DFE, dietary folate equivalent; ^(8)^ DRI, Dietary Reference Intakes; ^(9)^ DRI for ages 50 to 64; ^(10)^ nutrients without statistical significance are indicated in gray and italicized text.

**Table 6:** Summary of review studies on age-related eye diseases and carotenoids and antioxidants.

Disease Outcome	Nutrients Focusing on	Ref
ARC/AMD	Antioxidants: vitamins C, E, carotenoids	Chiu and Taylor (2007) [60]
Cataract	Antioxidants	Braakhuis et al. (2019) [62]
Cataract	Vitamin C, lutein/zeaxanthin, B vitamins, ω-3 fatty acids, multivitamins, and carbohydrates	Weikel et al. (2014) [63]
Cataract	Antioxidant interventions including vitamin C and vitamin C-based supplements	Lim et al. (2020) [64]
AMD	ω-3 fatty acids, lower-glycemic-index diets, and some carotenoids	Schleicher et al. (2013) [65]
Cataract, AMD, and glaucoma	Carotenoid, lower-glycemic-index diet, a healthy diet rich in fruits and vegetables	Kang et al. (2016) [66]
Cataract, AMD, and glaucoma	Dietary patterns and carbohydrates	Francisco et al. (2020) [67]

ARC, age-related cataract; AMD, age-related macular degeneration.

**Table 7:** Summary of related studies on age-related eye diseases and nutrients.

Study, Design	Nutrients Studied	Population (Age)	Disease Outcome	Key Findings	Ref.
Cross-sectional and prospective cohort study	Lutein, zeaxanthin, and vitamins B	3115 (55–80)	Cataract	The highest vitamins B_2_ (OR = 0.62 [0.43–0.90], *p* = 0.01), B_12_ (OR = 0.62 [0.43–0.88], *p* = 0.01), B_6_ (OR = 0.67 [0.45–0.99])	Glaser et al. (2015) [68]
Cross-sectional study	Total fruits, whole fruits, whole grains, and refined grains	6395 (over 30)	Cataract	Total fruits (OR = 0.95 [0.90–0.99], *p* = 0.027), whole fruits (OR = 0.95 [0.91–0.99], *p* = 0.016), whole grains (OR = 0.97 [0.94–0.100], *p* = 0.024), refined grains (OR = 0.96 [0.93–0.99], *p* = 0.002)	Zhou et al. (2022) [69]
Prospective cohort study	Vegetables, cruciferous vegetables	71,720 (45–74)	Cataract	Higher intake of vegetables for men [OR = 0.77 [0.59–1.01], *p* = 0.03), cruciferous vegetables for men (OR = 0.74 [0.57–0.96], *p* = 0.02), higher intake of vegetables for women (OR = 1.28 [1.06–1.53], *p* = 0.01)	Adachi et al. (2021) [70]
Hospital-based city cohort study	Na, coffee, a Western-style diet	41,067 (over 50)	ARC	High Na, Western-style diet, and low coffee intake elevated its risk	Jee et al. (2020) [71]
Cross-sectional cohort study	Vitamins A, B, C, and E, carotene	1155 (over 65)	Glaucoma	Vitamin B_2_ (OR = 0.39 [0.17–0.86], *p* = 0.019)	Coleman et al. (2008) [72]
Cross-sectional study	Mineral elements, calcium	5227 (mean age 70.20 ± 11.43)	AMD	Dietary calcium (OR = 0.68 [0.48–0.96], *p* = 0.029)	Chen and Chen (2023) [73]
Cohort study	Trans-unsaturated fat, ω-3 fatty acids, and olive oil	6734 (58–69)	AMD	Trans-fat (OR = 1.76 [0.92–3.37], *p* = 0.02), ω-3 (OR = 0.85 [ 0.71–1.02], *p* = 0.03; early AMD), olive oil (OR = 0.48 [0.22–1.04], *p* = 0.03; late AMD)	Chong et al. (2009) [74]
Multicenter case–control study	Carotenoids and vitamins A, C, and E	876 (55–80)	Advanced AMD	Higher carotenoid (OR = 0.57 [0.35−0.92], *p* = 0.02)	Seddon et al. (1994) [59]

AMD, age-related macular degeneration; ARC, age-related cataract.

## Data Availability

The datasets analyzed during the current study are available in the Korea Centers for Disease Control and Prevention database, which can be accessed through the following link: [Korea National Health and Nutrition Examination Survey] (https://knhanes.kdca.go.kr/knhanes/sub03/sub03_02_05.do, accessed on 25 August 2023). Registration for membership is required to utilize the raw data, and this privilege extends to both domestic and international researchers. It is important to note that the instructions for data access and the user manual are provided solely in Korean.
